# High-Heat Days and Presentations to Emergency Departments in Regional Victoria, Australia

**DOI:** 10.3390/ijerph19042131

**Published:** 2022-02-14

**Authors:** Jessie Adams, Susan Brumby, Kate Kloot, Tim Baker, Mohammadreza Mohebbi

**Affiliations:** 1 National Centre for Farmer Health, Western District Health Service, Hamilton, VIC 3300, Australia; susan.brumby@wdhs.net; 2 School of Medicine, Deakin University, Warrnambool, VIC 3280, Australia; kate.kloot@deakin.edu.au; 3 Centre for Rural Emergency Medicine, Deakin University, Warrnambool, VIC 3280, Australia; tim.baker@deakin.edu.au; 4 Biostatistics Unit, Faculty of Health, Deakin University, Burwood, VIC 3125, Australia; m.mohebbi@deakin.edu.au

**Keywords:** occupational health, farmers, extreme heat, heat-related illness, high-heat climate change, injury, heat exposure

## Abstract

Heat kills more Australians than any other natural disaster. Previous Australian research has identified increases in Emergency Department presentations in capital cities; however, little research has examined the effects of heat in rural/regional locations. This retrospective cohort study aimed to determine if Emergency Department (ED) presentations across the south-west region of Victoria, Australia, increased on high-heat days (1 February 2017 to 31 January 2020) using the Rural Acute Hospital Data Register (RAHDaR). The study also explored differences in presentations between farming towns and non-farming towns. High-heat days were defined as days over the 95th temperature percentile. International Statistical Classification of Diseases and Related Health Problems, Tenth Revision, Australian Modification (ICD-10-AM) codes associated with heat-related illness were identified from previous studies. As the region has a large agricultural sector, a framework was developed to identify towns estimated to have 70% or more of the population involved in farming. Overall, there were 61,631 presentations from individuals residing in the nine Local Government Areas. Of these presentations, 3064 (5.0%) were on days of high-heat, and 58,567 (95.0%) were of days of non-high-heat. Unlike previous metropolitan studies, ED presentations in rural south-west Victoria decrease on high-heat days. This decrease was more prominent in the farming cohort; a potential explanation for this may be behavioural adaption.

## 1. Introduction

The climate is changing, and it is predicted that extreme weather will be more frequent and intense [1]. Globally, the most recent five years have been the warmest on record. Internationally, research has shown an increase in illness, hospitalisations, and deaths during extreme weather events/days [2,3,4]. In Australia, 2019 was both the warmest and driest year on record [5]. 

Heat kills more Australians than any other natural disaster; between 1900 and 2011, extreme heat accounted for 55% (4555 deaths) of the total number of deaths due to natural hazards [6]. Heat-related illness occurs from combined environmental exposure, metabolic demands, and restricted cooling mechanisms, consequently causing the body to be unable to disperse heat adequately, resulting in thermoregulatory dysfunction [7]. According to Gauer and Meyers [7], an increase of approximately two degrees in core body temperature leads to heat-related illness which can vary in nature, from mild illness such as heat-rash, cramps, and heat exhaustion, through to heatstroke and death [7]. Strenuous exercise, poor physical fitness, exposure to high temperatures and humidity, lack of acclimatisation, dehydration, chronic disease, excessive clothing, and wearing protective equipment are all identified risk factors [8]. 

Research conducted in metropolitan areas of Australia have investigated the effects of exposure to high-heat days and high-heat events on human health. Similarly to international studies, reports showed increased ambulance call-outs and subsequent hospital admissions for heat-related conditions, including kidney disease, deterioration in mental health, heat-related injuries (such as sunburn and heat exhaustion), asthma, and heart disease [9,10,11,12,13]. Toloo, Yu, Aitken, FitzGerald, and Tong [12] concluded that as heat increased, the likelihood of attendance to Emergency Departments by all genders and age groups increased, with those presenting being more unwell. In Victoria, Australia, the 2009 heatwave saw a 12% increase in overall Emergency Department presentations, and 374 more deaths than expected for that time of the year [14]. 

The working environment has been identified as a key determinant in contributing to heat-related illness [4,12,15]. By exploring heat-related mortality and morbidity in outdoor workers in Los Angeles County, researchers from the Labour Occupational Safety and Health Program [4] concluded there was a strong association between the proportion of residents working in outdoor industries and rates of Emergency Department presentations during heat events. Additionally, there have been recent calls for the United States Occupational Safety and Health Association (OSHA) to issue federal heat standards that include water, shade, and rest breaks for farm workers [16]. In Victoria, Australia, there is no maximum temperature set under the Occupational Health and Safety Act 2004 or Occupational Health and Safety Regulations 2017 for a worker to stop working. Although, some workplace agreements cite 35 °C, where employers are required to consult with the union to minimise heat risks, and decide whether workers can continue to work safely [17]. Similarly, Bi, Williams, Loughnan, Lloyd, Hansen, Kjellstrom, Dear, and Saniotis [15] investigated the effects of extreme heat on health in Australia, and highlighted occupational heat stroke (heat stroke due to work) as an area for concern. 

Globally, agricultural workers have been identified as a high-risk group for suffering heat-related illness, and climate change will further increase this vulnerability [18]. Despite some coroner cases reporting deaths of agricultural workers (17 cases between 2000–2015), and data from Safe Work Australia [19] reporting 1174 (2009/10 to 2018/19) workers’ compensation claims from working in the heat, this has not been fully investigated in Australia. 

Additionally, Australian heat-related illness research has largely focused on metropolitan populations [15], despite mining, agriculture, construction, and emergency services, largely non-metropolitan occupations, having been identified as high-risk occupations for heat strain. There is also limited female data available for Australian occupational heat stress settings. This may in part be due to the industry (agriculture, mining, and construction) being male-dominated; however, according to Jay and Brotherhood [20], this disparity between male and female data far exceeds the ratio of males to females in these sectors. 

Therefore, the aim of this study was to determine and understand the prevalence and characteristics of heat-related illness in rural south-west Victoria (an agriculturally-based region) using the Rural Acute Hospital Data Registers (RAHDaR) from 1 February 2017 to 31 January 2020 between the warmer months of November and March, inclusive. It also aims to compare frequencies of Emergency Department presentations of individuals from farming towns and non-farming towns in rural south-west Victoria. The results from this study will assist in understanding the effects of high-heat days on the volume and types of Emergency Department attendances in rural/regional Australia. It also supports the importance of developing localised public health responses to high-heat days/events, whether in regional/rural Australia or internationally. 

## 2. Materials and Methods

**Figure 1:**
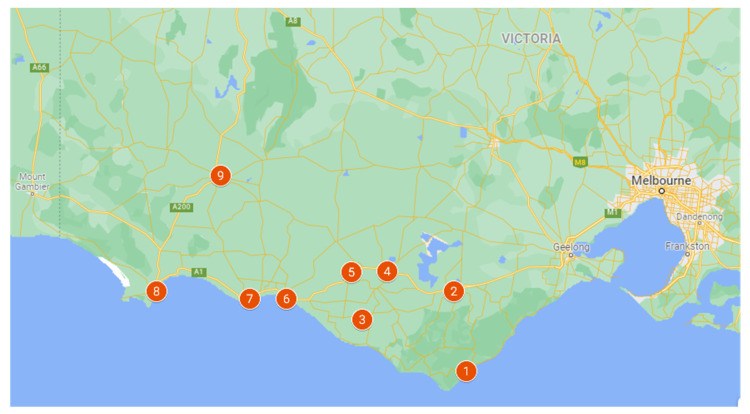
Map of the nine south-west Victorian hospitals (1. Otway Health; 2. Colac Area Health; 3. Timboon and District Healthcare Service; 4. South West Healthcare Camperdown; 5. Terang and Mortlake Health Service; 6. South West Healthcare Warrnambool; 7. Moyne Health Service; 8. Portland District Health; 9. Western District Health Service).

This was a cohort retrospective study utilising the Rural Acute Hospital Data Register (RAHDaR). The RAHDaR database collates detailed data on all presentations to the Emergency Department from every emergency care facility in south-west Victoria [21] (Figure 1); this is inclusive of very small rural facilities which run Urgent Care Centres (UCC). UCCs provide first-line emergency care; they can perform emergency resuscitation, stabilisation, and preparation of patient transfers to facilities that provide higher levels of care [22,23,24]. Though the Victorian Emergency Minimum Dataset (VEMD) includes mandatory-reported emergency presentations from larger Emergency Departments, RAHDaR also captures presentations at the lower-resourced south-west Victorian sites, such as the UCCs, and highlights as much as a 35% deficit in the data currently available via the government-reported dataset [25,26]. All data utilised in RAHDaR is Victorian Emergency Minimum Dataset (VEMD) and VEMD-equivalent data, and uses the International Statistical Classification of Diseases and Related Health Problems, Tenth Revision, Australian Modification (ICD-10-AM) [27]. The data are collected for epidemiological purposes, health service planning and coordination, policy assessment and formulation, clinical research and quality improvement, and patient management [21]. 

As this study aimed to investigate the prevalence and characteristics of heat-related illness, only the warmer months during the study period (1 February 2017 to 31 January 2020) were utilised. 

Ethics approval was sought from the South West Healthcare Human Research Ethics Committee (Ref: 2019 31) and Deakin University Human Research Ethics Committee (Ref: 2020-004).

### 2.1. Weather Data—Identification of Days of High-Heat 

There are no Australian or internationally agreed upon definitions of a high-heat day. Previous literature was explored to determine the best method to be used in this study. Days over the 95th and 99th temperature percentiles were identified. During data analysis, it was determined there were not enough days/presentations on days over the 99th percentile for it to be significant. Therefore, all days over the 95th temperature percentile were identified as days of high-heat, and in line with other research [12,28,29]. 

The weather stations closest to each of the nine acute hospitals were identified. Maximum daily temperature (highest temperature (°C) recorded in the 24 h to 9 a.m.) data were downloaded into Microsoft Excel (Microsoft, Washington, DC, USA) [30] from each weather station using the Bureau of Meteorology’s ‘Weather Station Directory’ [31]. All irrelevant data (colder months) were removed, with the months of interest remaining. The temperature percentiles were calculated using the days of the study period (November to March). Due to the varying climates between hospital locations and potential acclimitisation, the 95th temperature percentiles were calculated for each hospital. Table 1 shows the temperature cut-off points for each hospital. Days above these temperature cut-offs were classified as high-heat days, and the remaining days were categorised as days of non-high-heat.

### 2.2. ICD-10-AM Codes 

Although there are specific ICD-10-AM heat-related codes (T67.0 heatstroke and sunstroke, and T67.1 heat syncope), previous literature has highlighted that heat precipitates a large number of other conditions, resulting in heat-related codes rarely being used [27]. Diagnosis coding used in previous literature examining heat-related illness was reviewed. Many of these studies utilised diagnostic blocks (code groups) rather than specific diagnoses to identify heat-related illness; however, this may result in the over-identification of conditions included as heat-related [3,4,9,10,11,12,32,33,34]. In order to increase the accuracy, ICD-10-AM codes from within previously identified diagnostic blocks [3,4,9,10,11,12,32,33,34] were rated independently for relevance by two investigators, both experienced clinicians. These clinicians work within different areas of the rural healthcare system, and have different clinical backgrounds, minimising the potential for study bias in their code selection. All codes identified were compared, and any discrepancies were discussed (Table 2). 

In broad terms, the identified ICD-10-AM codes belonged to the following Major Diagnostic Blocks (code groups):

Any presentation with a diagnosis recorded as one of the 494 identified as heat-related were included in the study.

### 2.3. Farming and Non-Farming Town 

The south-west region of Victoria is located in the south-west corner of the state, and adjoins the South Australian border (see Figure 1). The region comprises the six local government areas of Colac Otway, Corangamite, Glenelg, Moyne, Southern Grampians, and Warrnambool, part of Surf Coast; and the centres of Colac, Hamilton, and Warrnambool. The region covers a total area of around 26,380 square kilometres or 11.6 per cent of Victoria’s total area [35,36]. Australian Bureau of Statistics (ABS) data indicate that in 2018–2019, there were 3554 farms in the Warrnambool and the south-west region, made up of dairy, beef and sheep grazing, and cropping. Agriculture, forestry, and fishing was the largest employment sector with 18,000 people, representing 28% of the region’s workforce. 

As RAHDaR does not collect employment details, it was determined that the most appropriate method to identify farmers was through town of residence. No previous work has identified towns with a high population percentage involved in agriculture. The Modified Monash Model is used to define a location as a city, rural, remote, or very remote in relation to their access to healthcare services [37]. Rural towns are identified as having a Modified Monash Model classification of MM3 or above. The classifications in this model are determined by a town’s population and distance to services, for example, a location is described as a MM3 if it is within 15 km road distance of a town with a population between 15,000 and 50,000 [37]. However, this model does not accurately identify whether a location is likely to have a large proportion of farming residents. For example, Apollo Bay is identified as MM5; however, agriculture is not identified in the top five industries of employed persons, with the top five industries making up 40% of those employed in Apollo Bay. Therefore, it cannot be assumed that towns identified as ‘rural towns’ by the Modified Monash Model are associated with agriculture. 

Consequently, a framework was developed to assist in identifying towns with the majority of the population involved in agriculture (Appendix A). It takes into consideration multiple factors when estimating the percentage of involvement in agriculture of a town’s population, including town population and main industries of employment as reported by the Australian Bureau of Statistics [38], and digital visualisation of the town to assess the presence of typical farming characteristics (and the assessment of clusters of houses) by researchers [39]. The Local Government Area top industries of employment and a town’s Modified Monash Model classification were also considered [36,38]. In this study, an area/town was classified as ‘farming’ if it was estimated that over 70% of the population were involved in agriculture. 

**Figure 2:**
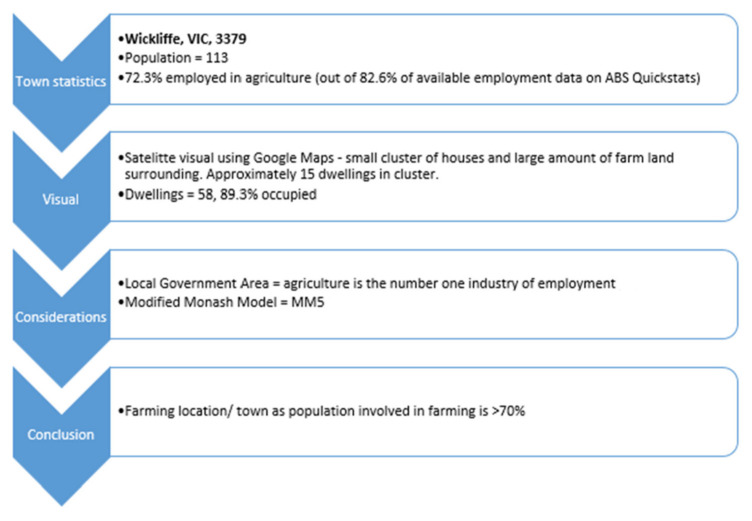
Example of using the farming framework for the location of Wickliffe to classify farming/non-farming locations [36,37,39,41].

The framework to identify farming towns underwent inter-rater reliability testing [40]. Three independent researchers estimated the percentage of those involved in farming in a sample of 110 towns using the framework as a guideline. Their estimations were compared to the original town classifications, and the calculated agreement was over 90%. This ensured reliability of the procedure. See Figure 2 as an example of using the decision framework. 

### 2.4. Data Extraction and Analysis

After identification of all the high-heat days during the study period, ICD-10 AM codes (related to heat-related illness) and farming towns were identified, and the relevant data were extracted from the RAHDaR database into a Microsoft Excel file. The Statistical Package for the Social Sciences (SPSS) version 27 (IBM, New York, NY, USA) was then used for data analysis [42]. Descriptive statistics (frequencies, percentages) were calculated to identify differences in presentations in extreme heat days and non-extreme heat days, as well as between the farming and non-farming populations. A logistic regression was computed using weighted data adjusting for age, gender, Modified Monash Model, and the Index of Socio-economic Advantage and Disadvantage to test statistical significance. Weighting was applied according to each LGA’s sex, age, and employment in agriculture statistics to ensure results were representative of the cohorts’ demographics. The rate of presentations per day was calculated using the average number of high-heat days (*n* = 23.8) and non-high-heat days (*n* = 429.4 days), and dividing it by total numbers of presentations. 

A logistic regression of the binary response variable of hospital presentations due to injury on the binary high-heat days independent variable with a sample size of 15,776 presentations achieves 90% power at a 0.05 significance level to detect a minimum odds ratio of 1.45.

## 3. Results

Overall, there were 76,457 presentations to the nine Emergency Departments (ED) and Urgent Care Centres in south-west Victoria during the 453 study days. A total of 3844 (5%) presented on days over the 95th temperature percentiles (as outlined in the Section 2), and 72,613 (95%) on days not classified as high-heat. A total of 19.4% (14,826) of the presentations belonged to individuals not residing in the nine Local Government Areas of south-west Victoria, and were removed from further analysis; therefore, the total number of presentations analysed was 61,631. 

Total populations for the farming and non-farming cohorts were calculated. The total population of the farming town cohort (of towns with approximately over 70% of the population involved in farming) was estimated to be 15,890 people, whereas the non-farming town population was 171,180 [38], resulting in a total target population for the study of 187,070. 

Table 3 shows the top six Major Diagnostic Blocks presenting to the Emergency Department during the study period. Injuries were the most common type of presentation in all groups. However, on high-heat days, there was a drop in the rate per day of injury. Though injuries decreased in both the farming and non-farming groups, the percentage difference was substantially greater in the farming population (41% vs. 25%). Additionally, musculoskeletal/connective tissue system illness also decreased in farmers on high-heat days (−40%). The results suggest that the rate of farmers presenting to the Emergency Department on high-heat days was lower for injuries (−41%), digestive system illness (−6%), musculoskeletal/connective tissue system illness (−40%), neurological system illness (−28%), and ‘other’ presentations (−16%) when compared to non-high-heat days. Conversely, circulatory system illness (+5%) and respiratory system illness (+12%) increased on days of high-heat in the farming cohort. In the non-farming group, the rate of presentations also increased for circulatory system illness (+8%) and ‘other’ presentations, which include alcohol and drug abuse (+2%), on high-heat days. Overall, total farmer presentations decreased by 21%, and non-farmers by 6.5%.

Table 4 demonstrates the characteristics of presentations to the south-west Victorian EDs on high and non-high-heat days between farmers and non-farmers. The average age of presentations was 42.3 years (±26.6). The farming cohort was slightly younger than the individuals presenting from non-farming areas. This difference in attendance was statistically significant on both days of high-heat (*p* = 0.025) and non-high-heat (*p* < 0.001). Overall, more females (*n* = 31,618, 51.3%) presented to ED than males (*n* = 30,013, 48.7%). However, there were more males (*n* = 2370, 52.2%) from farming areas presenting to ED than females from farming areas (*n* = 2170, 47.8%). On days of non-high-heat, there was a statistically significant difference (*p* < 0.001) between farmers and non-farmers and the gender presenting to ED. Farming males had higher presentations on days of high-heat (55.3%), whereas presentations of farming females were higher on non-high-heat days (47.9%). There was a statistically significant difference between MMM rankings between farmers and non-farmers on both high-heat (*p* < 0.001) and non-high-heat days (*p* < 0.001). Farming areas had a lower proportion of presentations from Modified Monash Model (MMM) 4 on high-heat and non-high-heat days, with the large majority of presentations from farming areas classified as MMM5. Similarly, the difference between farmers and non-farmers on high (*p* < 0.001) and non-high-heat days (*p* < 0.001) in the Index of Relative Socio-economic Advantage and Disadvantage (ISRAD) was significant. Presentations from non-farming areas were more likely to be associated with lower deciles of the ISRAD index, meaning higher socio-economic disadvantage, whereas farming presentations were mainly from middle ISRAD deciles. 

The majority of presentations arrived at Emergency Departments via a transport mode classified as ‘other’ (82.4%, *n* = 50,792), which includes private car or community/public transport/voluntary drivers, whereas 17.1% (*n* = 10,543) arrived in an ambulance service. There was a statistically significant difference between the arrival transport mode of farmers and non-farmers on both days of high-heat (*p* = 0.020) and non-high-heat days (*p* < 0.001), with the large majority of farmers arriving using ‘other’ transport modes. Most presentations were classified as ‘urgent’ (*n* = 19,689, 31.9%) and ‘semi urgent’ (*n* = 27,651, 44.9%). There was a statistically significant difference on both days of high- (*p* = 0.033) and non-high-heat (*p* < 0.001) between farmers and non-farmers and triage category. ‘Urgent’ presentations (Australian Triage Category 1–3) were higher in the non-farming areas on days of high-heat and non-high-heat, whereas ‘non-urgent’ presentations (Australian Triage Category 4–5) were higher in the farming group on both days of high- and non-high-heat [43]. The majority of cases were discharged home from ED (67.7%, *n* = 41,487). There was a statistically significant difference between the departure status of farmers and non-farmers on days on non-high-heat (*p* < 0.001). The results suggest that on high-heat days, more farmers were admitted to hospital (29.9%), and less were discharged home (61.9%) compared to days of non-high-heat (24.4% and 71.0%, respectively). 

Table 5 shows the results of logistic regression models comparing the prevalence of presentations to the Emergency Department and UCCs on heat and non-heat days between farmers and non-farmers after employing frequency weights on age, gender, and employment in agriculture at the LGA level. There was a significant difference in both the unadjusted and adjusted regressions. After adjusting for age, gender, Modified Monash Model (MMM), and the Index of Socio-economic Advantage and Disadvantage (ISRAD), farmers were 17% (95% CI: 0.73–0.95; *p* = 0.005) less likely to present to the Emergency Department on high-heat days compared to those from non-farming areas. 

## 4. Discussion

Globally, research has identified increased mortality and morbidity on high-heat days [44]. Similarly, previous Australian literature has recognised increases in ill health due to high-heat [9,12,45]. On the contrary, the results of this study have demonstrated that there is a general decrease in presentations to ED on days of high-heat in rural south-west Victoria. This was particularly the case in the farming population, as the results suggested farmers were 17% less likely to present to ED on high-heat days when compared to those from non-farming areas. This contrasts with Williams et al. [46], whom explored effects of heatwaves on morbidity and mortality across South Australia, and identified significantly increased presentations to ED during heatwaves in the city of Adelaide; however, the differences did vary in regional locations, which included mining, agricultural, and coastal areas. Unsurprisingly, as our study was completed in a rural area, the most common Major Diagnostic Block presenting to ED on both high and non-high-heat days was injury [47], with injury more common in rural and regional areas. However, on high-heat days, there was a marked decrease in injuries in both farmers and non-farmers, which is contradictory to other findings which typically see an increase in injuries on days of high-heat [9,48,49]. In accordance with previous research, circulatory system illness increased in both cohorts on days of high-heat [11,13]. Furthermore, respiratory system illness increased in the farming population on high-heat days. Research undertaken in Finland identified that health-related cardiorespiratory symptoms increased in people with pre-existing lung conditions, agricultural workers, pensioners, and the unemployed [50]. Khalaj, Lloyd, Sheppeard, and Dear [9] investigated the impacts on health of heat waves in five regions in the state of New South Wales, and identified the most common primary diagnoses for hospital admission were injury, circulatory system illness, and respiratory system illness. 

The results of this study suggest farmers are less likely overall to present to EDs than non-farmers. This finding was unexpected and contradictory to a Los Angeles County study exploring whether heat-related health outcomes are more prevalent in communities with higher proportions of construction, agricultural, and other outdoor industry workers—concluding that with each percentage increase in residents working in agriculture, a 10.9% increase in heat-related ED visits was reported [4]. Further, as the Australian farming population has been identified as an ageing workforce, and the risk of heat-related illness is higher for older people, it was anticipated that the farmers in this study population would be at even greater risk of heat-related illness due to the combination of older age and outdoor physical work [51]. Therefore, the results of this study suggest this farming population may be aware of the risks of working on high-heat days, and adapt their behaviours accordingly. Additionally, although not specifically developed for agriculture, Worksafe Victoria [52] and Safe Work Australia [53] provide guidance on working in heat. The Australian Centre for Agricultural Health and Safety [54] utilised the hierarchy of control to suggest methods of preventing heat-related illness while working on the farm. Furthermore, regional total fire ban days ban or severely restrict the use of vehicles, machinery, and mobile equipment, which may then contribute in reducing farm-related injuries [55]. However, it is hard to surmise if the reduction in the use of machinery and vehicles then lends itself to more physically challenging tasks, which could exacerbate and, thus, explain the increased cardiovascular and respiratory illnesses. 

Research has shown that exposure to hot environments reduces worker productivity, and increases the likelihood of work-related injuries (WRIs). However, these studies have been largely focused on metropolitan areas [15]. Behavioural adaptions to hot weather in farming/agriculture may include self-pacing, increased rest periods, performing more strenuous activities in the cooler parts of the day, working under shade or ventilated sheds, increased water consumption, avoiding dark surfaces, appropriate clothing/personal protective equipment, modifying tools or equipment to reduce physical demands, and monitoring environmental conditions [56,57,58]. Spector, Krenz, Rauser, and Bonauto [33] investigated agricultural workers’ heat-related illness claims in Washington State, identifying that the majority of claims were due to a lack of worker training and a lack of a heat safety plan. This may not be as prevalent in the Australian farming context, as 99% of farms in Australia are family owned and operated, and as a result, farmers may be able to adapt their behaviours to avoid outdoor/physical work in extreme heat [59] In the USA, workers carried out, on average, one hour less work per day when temperatures exceeded 37 °C (compared with days in which the temperature was below 30 °C), as workers self-paced to maintain thermal comfort [60]. Heat-related health risks increase when work is ‘externally paced’, such as through production quotas, peer pressure, and remuneration by output, factors found in fruit picking and sheep shearing occupations [61]. The European Agency for Safety and Health at Work [58] suggested that modern technology could further assist in preventing heat-related illness in farmers, such as using smart watches or similar devices to monitor vital signs (e.g., heart beat and blood pressure), which could prevent serious illness, particularly circulatory system illness. Family farming, by its very nature, does allow for self-pacing, and this may help to explain the reductions in ED presentations on high-heat days from farmers, and should be promoted in outdoor working industries globally. 

The results of this study highlight the importance of considering context in regard to heat-related illness, as local factors, including climate, demographics, and acclimatisation/adaptive capacity can play a large role; it is not a one size fits all [62]. It has been suggested that the impacts of heatwaves in metropolitan areas are more severe, as the built environment retains more heat [63,64]. Conversely, characteristics commonly present in regional/rural populations, including occupational (higher proportions of outdoor workers) and socio-demographic (e.g., lower socio-economic status, ageing population) factors, present increased risks for heat-related illness [3,46]. Sun, Weinberger, Nori-Sarma, Sprangler, Sun, Dominici, and Wellenius [62] emphasised the importance of local public health preparedness for specific locally defined temperature thresholds, as communities are acclimatised to different climates. This may include identifying local at-risk populations, increasing public awareness of heat-related health risks, heat illness prevention education, identification of local hazards and potential exposures, and the communities’ and local healthcare facilities’ adaptive capacity for potential increases in demand [28,62]. On a local level, the Victorian Government, Australia, currently issues heath alerts to organisations and the public for days over 30 °C [65]. More contextual public health service messages (e.g., to agricultural workers [58]) could be released through local organisations and social media to remind people of the increased risk of illness on high-heat days, specifically, cardiovascular and respiratory illness in this study population, as well as suggestions for reducing the risk, and acting quickly if unwell. Health care workers can also provide relevant information on preventing heat-related illness (e.g., the exacerbation of cardiovascular disease and respiratory illnesses, particularly to farming populations). 

As the climate is changing, and high-heat days are likely to become more frequent and intense, it is important to understand protective factors for heat-related illness [66]. Currently, the farming/agriculture industry experiences high rates of injury and workplace fatalities [67]; however, as the results of this study demonstrate, on high-heat days, there is a large reduction of farmers presenting to ED and UCCs with injury. This is the first study to utilise the farming decision framework. The study was strengthened by the recruitment of all presentations (of south-west Victorian LGA residents) to the Emergency Departments during the study period to avoid selection bias. As the RAHDaR database does not collect occupation status, this decision framework allows for the identification of these two cohorts (farming and non-farming) to allow for a closer comparison of the behaviours and use of services. The authors acknowledge that the decision framework may miss some farm workers who reside in non-farming areas and regional towns or cities, and this is identified as a limitation of the decision framework. However, this study does highlight the value of collecting occupation data at both Emergency Departments and UCCs, as this is currently not collected [68]. It also calls attention to the importance of understanding local context before initiating a public health heat response. 

## 5. Conclusions

This is the first study to examine presentations to ED and UCCs in a rural Australian setting, and explore farming populations’ use of these services. This study confirms that unlike previous metropolitan studies, there is an overall decrease in presentations to ED on high-heat days in rural Victoria. Farmers are less likely than non-farmers to present on high-heat days overall, and with a significant reduction of injuries and musculoskeletal/connective tissue system illness in comparison to non-farmers. This suggests farmers in this cohort are aware of the risks of working/engaging in high-intensity activities on high-heat days, and adjust their behaviours accordingly. Those from farming towns that do attend, however, are more likely to be admitted for cardiovascular and respiratory type illness. The model used in this study could be replicated in other regions to address gaps in rural data, and better understand the patterns of presentations to Emergency Departments. This would enable better preparation, and possibly prevent the predicted presentations on high-heat days in rural and agricultural communities. Further investigation is needed into the effects of consecutive high-heat days, and the day following a high-heat day.

## Figures and Tables

**Table 1:** Hospital 95th percentile temperature cut-off points (°C).

Victorian Bureau of Meteorology Weather Station	Acute Hospital	Temperature Cut-off Point (95th Temperature Percentile)
Cape Otway Lighthouse	Otway Health	33.2 °C
Mortlake Racecourse	Timboon and District Health Service Terang and Mortlake Health Service South West Healthcare—Camperdown	36.5 °C
Warrnambool Airport	South West Healthcare—Warrnambool	34.1 °C
Mount Gellibrand	Colac Area Health	36.7 °C
Portland Cashmore Airport	Portland District Health	33.0 °C
Hamilton Airport	Western District Health Service—Hamilton	36.8 °C
Port Fairy	Moyne Health Service	34.4 °C

**Table 2:** Number of ICD-10-AM codes included under each Major Diagnostic Block.

Major Diagnostic Block	Number of Included Diagnoses
Injury, single site, major	186
Circulatory system illness	46
Alcohol/drug abuse and alcohol-/drug-induced mental disorders	36
Poisoning	30
Respiratory system illness	28
Injury, single site, minor	28
Psychiatric illness	26
Urological system illness	19
Illness of the ear, nose, and throat	19
Neurological system illness	17
Endocrine, nutritional, and metabolic system illness	14
Other presentation (not listed elsewhere)	11
Injury, multiple sites	11
Allergy	9
System infection/parasites	4
Blood/immune system illness	3
Illness of skin, subcutaneous tissue, breast	2
Musculoskeletal/connective tissue system illness	2
Hepatobiliary system illness	2
Digestive system illness	1
Grand Total	494

**Table 3:** Major Diagnostic Blocks presenting to the Emergency Department on high/non-high-heat days in farming and non-farming areas.

Major Diagnostic Blocks	Overall Presentations	Farming Areas				Non-Farming Areas			
		High-Heat Days *n* (Rate per Day)	Non-High-Heat Days *n* (Rate per Day)	Difference per Day	% Difference on High-Heat Days (Rounded)	High-Heat Days *n* (Rate per Day)	Non-High-Heat Days *n* (Rate per Day)	Difference per Day	% Difference on High-Heat Days (Rounded)
Injury (multiple sites, single site major, single site minor)	15,776	41 (1.72)	1251 (2.91)	−1.2	−41%	579 (24.33)	13,905 (32.38)	−8.05	−25%
Digestive system illness	6630	24 (1.01)	459 (1.07)	−0.06	−6%	305 (12.82)	5842 (13.61)	−0.79	−6%
Circulatory system illness	5051	16 (0.67)	274 (0.64)	0.03	5%	268 (11.26)	4493 (10.46)	0.80	8%
Respiratory system illness	3877	17 (0.71)	274 (0.64)	0.08	12%	187 (7.86)	3399 (7.92)	−0.06	−1%
Musculoskeletal/connective tissue system illness	3215	7 (0.29)	212 (0.49)	−0.20	−40%	150 (6.30)	2846 (6.63)	−0.33	−5%
Neurological system illness	3089	8 (0.34)	201 (0.47)	−0.13	−28%	149 (6.26)	2731 (6.36)	−0.10	−2%
Other (alcohol/drug abuse and alcohol-/drug-induced mental disorders; allergy; blood/immune system illness; endocrine, nutritional, and metabolic system illness; gynaecological illness; hepatobiliary system illness; illness of other and unknown symptoms; illness of the eyes; injury, multiple sites; male reproductive system illness; newborn/neonate illness; obstetric illness; poisoning; psychiatric illness; social problem; other presentation *)	20,780	71 (2.98)	1528 (3.56)	−0.58	−16%	1024 (43.03)	18,157 (42.28)	0.74	2%
Total	58,418	184 (7.73)	4199 (9.78)	−2.05	−21%	2662 (111.85)	51,373 (119.64)	−7.79	−7%

* Other presentations, *n* = 5708; missing cases, *n* = 3213. The rate per day was calculated using the average number of days of high-heat (*n* = 23.8 days) and non-high-heat days (*n* = 429.4 days).

**Table 4:** Characteristics of presentations to the Emergency Departments across the south-west.

Characteristics of ED Presentations	Overall	Days of Extreme Heat				Days of Non-Extreme Heat			
	*N* (%)	Farming Areas *n* (%)	Non-Farming Areas *n* (%)	DF	Sig (*p*-Value)	Farming Areas *n* (%)	Non-Farming Areas *n* (%)	DF	Sig (*p*-Value)
**Age**									
Mean (years)	42.3	39.4	43.7	1	0.025	38.6	42.6	1	<0.001
**Gender**									
Male	30,013 (48.7%)	110 (55.3%)	1399 (48.8%)	1	0.79	2260 (52.1%)	26,244 (48.4%)	1	<0.001
Female	31,618 (51.3%)	89 (44.7%)	1466 (51.2%)			2081 (47.9%)	27,982 (51.6%)		
Arrival Transport Mode *				1	0.020			1	<0.001
Ambulance Service (Air Ambulance, Ambulance Service, Helicopter, Road Ambulance, police vehicle)	10,543 (17.1%)	24 (12.3%)	540 (19.1%)			536 (12.4%)	9443 (17.5%)		
Other (Private car, community/public transport/voluntary drivers)	50,792 (82.4%)	171 (87.7%)	2293 (80.9%)			3372 (87.6%)	44,556 (82.5%)		
Triage Category **				3	0.033			3	<0.001
Resuscitation and Emergency	5681 (9.2%)	20 (10.0%)	283 (9.9%)			374 (8.6%)	5004 (9.2%)		
Urgent	19,689 (31.9%)	61 (30.8%)	1008 (35.2%)			1244 (28.7%)	17,376 (32.1%)		
Semi Urgent	27,651 (44.9%)	79 (39.9%)	1224 (42.8%)			1966 (45.3%)	24,382 (45.0%)		
Non-Urgent	8589 (13.9%)	38 (19.2%)	345 (12.1%)			756 (17.4%)	7450 (13.7%)		
Departure Status ***				2	0.661			2	<0.001
Admitted	15,754 (25.7%)	58 (29.9%)	759 (26.9%)			1052 (24.4%)	13,885 (25.7%)		
Discharged home	41,487 (67.7%)	120 (61.9%)	1818 (64.3%)			3059 (71.0%)	36,490 (67.6%)		
Left at own risk/after clinical advice	3981 (6.5%)	16 (8.2%)	245 (8.7%)			192 (4.5%)	3528 (6.5%)		
**Modified Monash Model**				1	<0.001			1	<0.001
MMM4 or less	40,412 (65.6%)	13 (6.5%)	2037 (71.1%)			222 (5.1%)	38,140 (70.3%)		
MMM5	21,219 (34.4%)	186 (93.5%)	828 (28.9%)			4119 (94.9%)	16,086 (29.7%)		
**Index of Relative Socio-economic Advantage and Disadvantage (ISRAD)**				1	<0.001			1	<0.001
2–4	46,864 (76.0%)	64 (32.2%)	2306 (80.5%)			1329 (30.6%)	43,165 (79.6%)		
5–7	12,730 (20.7%)	124 (62.3%)	468 (16.3%)			2693 (62.0%)	9445 (17.4%)		
8–10	2037 (3.3%)	11 (5.5%)	91 (3.2%)			319 (7.3%)	1616 (3.0%)		

* ‘Undertaker’ (*n* = 3) presentations removed. ‘Police vehicle’ (*n* = 252) in ambulance service, as these are typically mental health or intoxicated patients. Missing cases, *n* = 293; ** Missing cases, *n* = 21; *** Correctional/custodial facility (*n* = 45), dead on arrival (*n* = 8), and died within ED (*n* = 17) removed as numbers too low.

**Table 5:** Logistic regression comparing prevalence of presentations to the Emergency Department on heat/non-heat days with farming status.

Model	Chi-Square	Degrees of Freedom	*p*-Value	Odds Ratio	95% Confidence Intervals	
					Lower	Upper
Unadjusted	18.43	1	<0.001	0.79	0.71	0.88
Adjusted Gender and Age *	17.65	1	<0.001	0.80	0.72	0.89
Confounder Adjusted **	7.78	1	0.005	0.83	0.73	0.95

* Adjusted for age and gender; ** Adjusted for age, gender, Modified Monash Model (MMM), and Index of Relative Socio-economic Advantage and Disadvantage (ISRAD).

**Figure A1:**
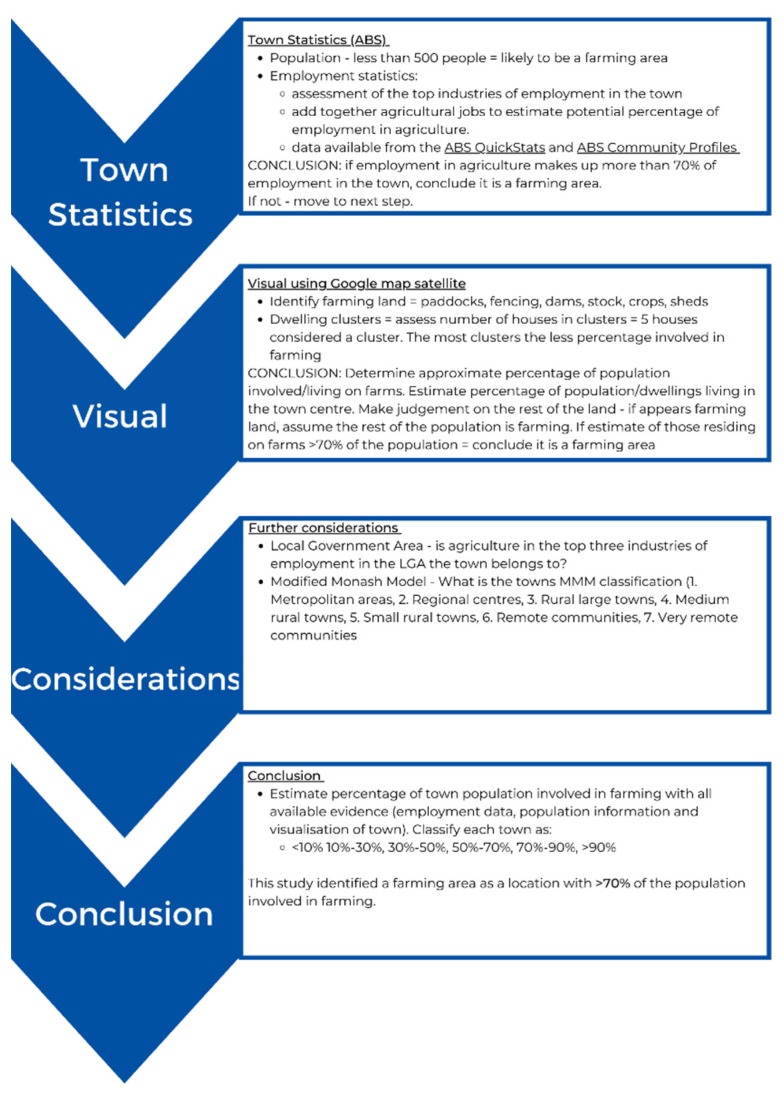
Framework for determining farming populations.
